# Evaluation of the capability of a real-time PCR assay to detect mycoplasma in advanced therapy medicinal products

**DOI:** 10.1186/1753-6561-9-S9-P72

**Published:** 2015-12-14

**Authors:** Alexandra Scholz, Stephanie Schweizer, Dirk Vollenbroich

**Affiliations:** 1 Sartorius Stedim Biotech GmbH, D-37079 Goettingen, Germany; 2 Minerva Biolabs GmbH, D-12555 Berlin, Germany

## Background

Advanced therapy medicinal products (ATMPs) are new medical products based on genes (gene therapy), cells (cell therapy) and tissues (tissue engineering).

Mycoplasmas are among the world's smallest bacteria capable of independent reproduction. They belong to the class of Mollicutes, have a very slow and parasitic growth and can cause human infections. Mycoplasma contaminations of ATMPs can arise from unsterile source material of different human origin, starting materials or the complex manufacturing process. The traditional growth-based detection method requires a cultivation time of at least 28 days before a contamination can be ruled out with certainty [1]. But shelf lives of final ATMPs are often extremely short compared to classical drugs (24 - 48 h, sometimes only a few hours). That is why the official culture method or indicator cell method are not suitable. Furthermore ATMP sample amounts are usually limited and of great "value".

Nucleic Acid Amplification Techniques (NAT) allow reducing time to result to just hours and only small sample amount are necessary to generate highly sensitive results.

A Mycoplasma Real-time PCR kit was designed especially for the detection of Mollicutes (Mycoplasma, Acholeplasma, Spiroplasma) contamination in ATMPs and cell cultures by using the cells itself, cell culture supernatant or a defined mixture as test material. An acceptable sample volume in respect of the expensive, unique and limited sample is processed for Mycoplasma detection. In this studies the robustness of the kit was demonstrated by testing 20 randomly selected Advanced Therapy Medicinal Products from samples submitted by customers for Mycoplasma detection. These tests were done as part of the kit validation. All selected ATMPs were tested negative for Mycoplasma and could therefore be used as spiking matrices for the intended studies. These 20 different products were spiked with 10 CFU/ml of Mycoplasma fermentans. Each spiked ATMP matrix was then subjected to a DNA isolation process and tested by Real-time PCR.

All 20 randomly selected ATMPs out of 20 in total were tested positive in duplicate and fulfilled the acceptance criterion. These results confirmed the suitability and the robustness of the designed Mycoplasma Real-time PCR kit for ATMP testing.

## Materials and methods

**Figure 1:**
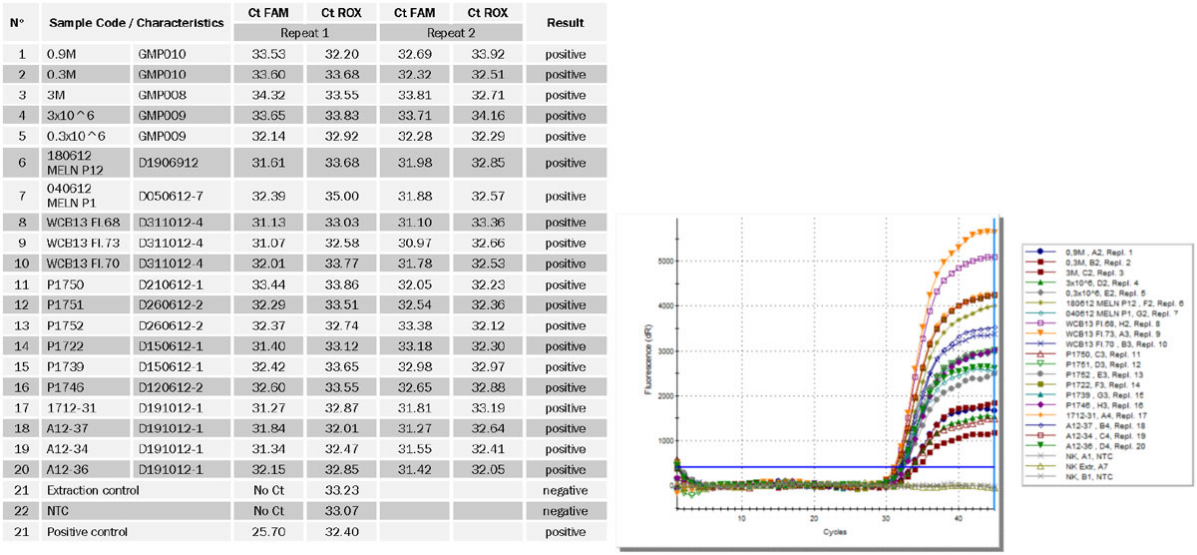
**Left: Results of 20 ATMP matrices spiked with *M***. fermentans (10 CFU/ml); FAM channel detects *Mycoplasma *DNA, ROX channel visualizes the internal inhibition control reaction; Right: Amplifications curves (FAM channel) of 20 ATMP matrices spiked with *M. fermentans *(10 CFU/ml)

Two replicates of 20 randomly selected Mycoplasma negative ATMP samples (see Fig. 1) were spiked with Mycoplasma fermentans at a final concentration of 10 CFU/ml. 200 µl of each sample were used for silica-membrane based DNA extraction followed by Real-time PCR detection on the cycler MxPro 3005P. In parallel unspiked sample material was processed as a negative extraction control. After Real-time PCR detection Ct values were determined and result interpretation was done.

## Results

All 20 randomly selected ATMPs out of 20 in total were tested positive in duplicate and fulfilled the acceptance criterion (Ct values < 40). None of the matrices showed inhibitory effects in the ROX channel (Fig. 1). Mean Ct values were 32.3 ± 0.9 for FAM and 33.0 ± 0.7 for ROX.

The Real-time PCR used is a multiplex assay which is able to detect Mollicutes with a FAM-labeled probe and an internal control DNA plasmid with a ROX-labeled probe. The ROX channel is used to check whether the reaction is inhibited due to matrix effects for example. A positive signal in the ROX channel indicates the absence of inhibitory effects and a successful amplification reaction.

## Conclusions

These studies provided detailed information about the suitability and robustness of the kit by reflecting influences of the sample matrices frequently used for manufacturing of Advanced Therapy Medicinal Products and in cell culture technology in general. M. fermentans was easily detectable at the spiked concentration of 10 CFU/ml even in highly complex sample matrices.
